# Anxiety and Sleep Problems of College Students During the Outbreak of COVID-19

**DOI:** 10.3389/fpsyt.2020.588693

**Published:** 2020-11-23

**Authors:** Xing Wang, Hongguang Chen, Ling Liu, Yuan Liu, Nan Zhang, Zhenghai Sun, Qing Lou, Weichun Ge, Bo Hu, Mengqian Li

**Affiliations:** ^1^Nanchang University Institute of Life Science, School of Life Sciences (Nanchang University), Nanchang, China; ^2^Peking University Sixth Hospital, Peking University Institute of Mental Health, NHC Key Laboratory of Mental Health (Peking University), National Clinical Research Center for Mental Disorders (Peking University Sixth Hospital), Beijing, China; ^3^Department of Psychosomatic Medicine, The First Affiliated Hospital of Nanchang University, Nanchang, China; ^4^College of Humanities, Jiangxi University of Traditional Chinese Medicine, Nanchang, China; ^5^Department of Public Administration, Nanchang University, Nanchang, China; ^6^Mental Health School of Qiqihar Medical University, Qiqihar, China; ^7^Yichun University, Yichun, China

**Keywords:** COVID-19, college students, psychological health, correlator, psychopathology

## Abstract

This study aimed to explore the psychological situation and the influence of the outbreak of COVID-19 on college students. An online questionnaire survey was conducted among 3,092 Chinese college students who were quarantined at home as a result of the COVID-19 pandemic. The survey tools included the Generalized Anxiety Disorder 7-Item Scale (GAD-7), the Perceived Stress Scale (PSS-10), and the Self-Rating Scale of Sleep (SRSS). Of all the respondents, the prevalence of anxiety symptoms, sleep problems, any of the two, and both of the two, were 16.8, 13.5, 25.1, and 5.3%, respectively. Of the participants, 43.7% of the college students had higher perceived stress. Factors associated with anxiety symptoms included reading the daily news with higher frequency (1–3 times; 4–7 times; more than 7 times), having sleep problems, higher stress, and carelessness with the number of remaining masks. Factors associated with sleep problems included postgraduates, reading the news with higher frequency daily (1–3 times), the frequency of going out per week (1–3 times), having anxiety symptoms and higher stress. Factors associated with higher perceived stress included reading the daily news with higher frequency (4–7 times), anxiety about the number of remaining masks (1–10; more than 20), having anxiety symptoms, and having sleep problems. The prevalence of anxiety symptoms, sleep problems, and higher perceived stress among college students was high during the COVID-19 outbreak. Particular attention should be paid to psychological support for college students quarantined at home, especially those at high risk of psychological problems.

## Introduction

Since December 2019, the emergence of COVID-19 has been continuously reported throughout China. Compared to SARS or MERS, COVID-19 is more infectious and spreads faster (1). As of June 3, 2020, more than 84,000 cases had been confirmed in China (2). The cumulative number of diagnoses abroad had exceeded 6 million (2). According to the statistics of the United Nations Educational, Scientific, and Cultural Organization, the COVID-19 epidemic caused 146 countries to suspend school, and the number of students influenced has reached 1.18 billion (3). Similar to SARS, the COVID-19 outbreak also had a profound impact on people's psychosocial and mental health (4). There have been reports on the psychological status of the general population (5), children (6), medical staff (7), international Chinese students (8), maternal populations (9), and the elderly (10) during the epidemic. In contrast to other populations, college students are more likely to be affected by the COVID-19 epidemic, experiencing uncertainty and abrupt disruption of the semester (11). Meanwhile, college students are one of the special social groups that have attracted much attention. However, there has been little research on the psychological status of college students during the COVID-19 epidemic to date. It was reported that anxiety symptoms and sleep problems might be more prominent in the early stages of the epidemic (12). Thus, the purpose of this study was to assess the prevalence of anxiety and sleep problems and associated factors caused by the COVID-19 pandemic.

## Methods

### Study Design

This non-probability sampling survey was conducted among college students in China from February 21 to March 7, 2020. The survey used online questionnaires, which were administered through a web-based survey platform. During the survey, a WeChat QR code with a questionnaire link was sent to WeChat groups at Universities located in 31 provinces and cities across the country. Overseas students were excluded. These questionnaires were completed once per interviewee. The research was approved by the Research Ethics Board at the First Affiliated Hospital of Nanchang University (approval number: 050) and electronic informed consent was obtained from each subject who took part.

### Questionnaire

The questionnaire was divided into two parts. The first part collected the basic information of the participants, including the participants' education, gender, and personal protective behaviors during the epidemic. The second part investigated the participants' psychosomatic symptoms during the epidemic, including anxiety, stress, and sleep conditions.

#### Personal Health Status and Behaviors

This part included personal health status (patients diagnosed with COVID-19/suspected contact/healthy people), the number of times daily they read about the COVID-19 epidemic in the news, the number of times they went out per week, the type of masks they used, the number of remaining masks they had, and the most common network behavior during the epidemic period.

#### Generalized Anxiety Disorder 7-Item (GAD-7) Scale

The Generalized Anxiety Disorder Scale was compiled by Spitzer (13) and is used for screening generalized anxiety and assessment of symptoms severity. It consists of seven items and is used to understand how long respondents are troubled by seven problems, including “difficult to relax” and “excessively worried about various problems” in the past 2 weeks. GAD-7 has proven to be a reliable tool for measuring anxiety (14). In this study, cases with anxiety symptoms were defined as a total score of GAD-7 ≥ 5 (9).

#### Perceived Stress Scale (PSS-10)

The Perceived Stress Scale was a self-assessment scale compiled by Cohen (15) and is used to assess the degree of stress experienced by an individual in the past month. PSS-10 was used to assess situations that individuals find it difficult to control, difficult to predict, or when they feel overwhelmed. The questionnaire had a total of 10 items, and each item was divided into 0 = never to 4 = very common with a total of 5 grades. Six items in PSS-10 were considered negative (items 1, 2, 3, 6, 9, and 10), which assessed the level of pain. The other four items were positive (items 4, 5, 7, and 8), reflected people's views on the capabilities of stressors. When calculating the total score of PSS-10, the positive entries were reverse coded (15, 16). The higher the score on this scale, the higher the level of perceived stress. In this study, higher perceived stress was defined as a total score of PSS-10 ≥ 14.

#### Self-Rating Scale of Sleep (SRSS)

Self-Rating Scale of Sleep is suitable for screening sleep problems in different populations (17). It can also be used to compare the effects of sleep problems before and after treatment (18). This scale has good reliability and validity (19). The higher the score on this scale, the worse the sleep problems. In this study, cases with sleep problems were defined as a total score of SRSS ≥ 23 (17).

### Statistical Analysis

A Chi-square test was used to compare the characteristics of distribution for both anxiety and sleep problems. Binary logistic regression analysis was performed to screen the factors associated with anxiety and sleep problems and calculate the ORs (odds ratios) and a 95% CI (confidence interval). Statistical tests were two-tailed with *p* < 0.05. The database was constructed by EpiDate3.1 and analyzed by SPSS 25.0.

## Results

### Demographic Characteristics of the Respondents

Among all the 3,092 investigated college students, 33.6% were male and 66.4% were female. The respondents were located in 31 provinces and cities across the country, of which 34.8% were from Jiangxi province, located in the southern region of China, 14.5% from Heilongjiang Province, located in the northern region of China, 5.1% from Hubei Province, located in the central region of China and the remaining 45.6% were from other provinces. There were 87.9% undergraduates and 12.1% postgraduates (not including Ph.D. students). The most popular online behavior during the period of the epidemic was playing games, which accounted for 19.6% of the total.

### The Prevalence of Anxiety Symptoms, Sleep Problems, and Perceived Higher Stress Among College Students

Among the investigated college students, the prevalence of anxiety symptoms, sleep problems, anxiety symptoms or sleep problems, and anxiety symptoms and sleep problems were 16.8, 13.5, 25.1, and 5.3%, respectively. Overall, 43.7% of college students had higher perceived stress.

The prevalence of anxiety symptoms was significantly higher in college students with the following characteristics: postgraduates (22.4 vs. 16.1%) and those who read the news with higher frequency daily [None (9.8%); 1–3 times (15.1%); 4–7 times (19.0%); more than 7 times (29.6%)]. In terms of wearing a mask, these experienced anxiety when not wearing a mask (22.1%); wearing a disposable medical mask (15.5%); wearing an N95 mask or medical protective mask (14.9%); when wearing other common masks (19.1%), and when they had few remaining masks [0 (22.0%); 1–10 (17.0%); 10–20 (16.6%); had more than 20 masks (15.2%); and those who did not know how many remaining masks they had (9.5%)]. The students also experienced anxiety when they were having sleep problems (39.1 vs. 13.4%) and higher perceived stress (27.3 vs. 8.7%) (Table 1).

**Table 1:** Prevalence of anxiety symptoms, sleep problems, and perceived stress among college students^†^.

**Characteristic**	**Group**	***N***	**Anxiety symptoms % (95%CI)**	**Sleep problems % (95%CI)**	**Any of the two % (95%CI)**	**Both of the two % (95%CI)**	**Perceived stress % (95%CI)**
Gender	Male	1,038	16.3 (14.0–18.5)	14.0 (11.9–16.1)	25.0 (22.4–27.7)	5.2 (3.8–6.6)	45.5 (42.4–48.5)
	Female	2,054	17.1 (15.5–18.8)	13.2 (11.8–14.7)	25.1 (23.2–26.9)	5.3 (4.3–6.3)	42.8 (40.7–45.0)
	*P*		0.548	0.576	0.988	0.902	0.164
Education	Bachelor	2,717	16.1 (14.7–17.5)	12.6 (11.3–13.8)	23.8 (22.2–25.5)	4.8 (4.0–5.6)	43.4 (41.6–45.3)
	Postgraduate	375	22.4 (18.2–26.6)	20.0 (15.9–24.1)	33.9 (29.1–38.7)	8.5 (5.7–11.4)	45.9 (40.8–50.9)
	*P*		0.002	<0.001	<0.001	0.003	0.373
Frequency of daily news reading	None	133	9.8 (4.7–14.9)	21.1 (14.0–28.1)	25.6 (18.1–33.1)	5.3 (1.4–9.1)	53.4 (44.8–62.0)
	1–3 times	2,192	15.1 (13.6–16.6)	11.7 (10.4–13.1)	22.8 (21.1–24.6)	4.0 (3.2–4.8)	42.7 (40.6–44.8)
	4–7 times	463	19.0 (15.4–22.6)	14.9 (11.6–18.2)	27.2 (23.1–31.3)	6.7 (4.4–9.0)	41.9 (37.4–46.4)
	More than 7 times	304	29.6 (24.4–34.8)	20.7 (16.1–25.3)	37.8 (32.3–43.3)	12.5 (8.8–16.2)	49.7 (44.0–55.3)
	*P*		<0.001	<0.001	<0.001	<0.001	0.012
Frequency of going out per week	None	1,900	16.1 (14.4–17.7)	12.3 (10.8–13.7)	23.3 (21.4–25.2)	5.0 (4.0–6.0)	43.0 (40.8–45.2)
	1–3 times	1,062	17.5 (15.2–19.8)	14.9 (12.7–17.0)	27.1 (24.4–29.8)	5.3 (3.9–6.6)	44.1 (41.1–47.1)
	More than 3 times	130	23.1 (15.7–30.4)	20.0 (13.0–27.0)	33.8 (25.6–42.1)	9.2 (4.2–14.3)	51.5 (42.8–60.2)
	*P*		0.091	0.012	0.004	0.113	0.159
Mask type	Not wearing a mask	335	22.1 (17.6–26.6)	18.5 (14.3–22.7)	30.4 (25.5–35.4)	10.1 (6.9–13.4)	54.0 (48.7–59.4)
	Disposable medical mask	1,754	15.5 (13.8–17.2)	11.7 (10.2–13.2)	23.4 (21.4–25.4)	3.8 (2.9–4.7)	41.4 (39.1–43.7)
	N95 mask or medical protective mask	402	14.9 (11.4–18.4)	15.4 (11.9–19.0)	24.9 (20.6–29.1)	5.5 (3.2–7.7)	42.5 (37.7–47.4)
	Other common masks	601	19.1 (16.0–22.3)	14.6 (11.8–17.5)	27.1 (23.6–30.7)	6.7 (4.7–8.7)	45.6 (41.6–49.6)
	*P*		0.007	0.003	0.027	<0.001	<0.001
Remaining mask	0	441	22.0 (18.1–25.9)	17.2 (13.7–20.8)	29.5 (25.2–33.8)	9.8 (7.0–12.5)	53.5 (48.8–58.2)
	1–10	1,427	17.0 (15.0–18.9)	13.2 (11.5–15.0)	25.2 (23.0–27.5)	5.0 (3.8–6.1)	41.3 (38.8–43.9)
	10–20	440	16.6 (13.1–20.1)	11.6 (8.6–14.6)	24.1 (20.1–28.1)	4.1 (2.2–5.9)	46.6 (41.9–51.3)
	More than 20	605	15.2 (12.3–18.1)	12.2 (9.6–14.8)	23.3 (19.9–26.7)	4.1 (2.5–5.7)	37.7 (33.8–41.6)
	Careless the number of remaining masks	179	9.5 (5.2–13.8)	15.1 (9.8–20.4)	21.2 (15.2–27.3)	3.4 (0.7–6.0)	52.0 (44.6–59.3)
	*P*		0.002	0.094	0.125	<0.001	<0.001
Network behavior	Others	2,485	16.7 (15.3–18.2)	13.4 (12.1–14.8)	25.1 (23.4–26.8)	5.1 (4.2–6.0)	42.9 (41.0–44.9)
	Online games	607	17.3 (14.3–20.3)	13.7 (10.9–16.4)	25.0 (21.6–28.5)	5.9 (4.0–7.8)	47.0 (43.0–50.9)
	*P*		0.742	0.880	0.988	0.418	0.074
Stress	Normal	1,740	8.7 (7.4–10.1)	7.6 (6.4–8.9)	15.0 (13.3–16.7)	1.4 (0.8–1.9)	–
	Higher^a^	1,352	27.3 (24.9–29.7)	21.0 (18.8–23.2)	38.0 (35.4–40.6)	10.3 (8.7–11.9)	–
	*P*		<0.001	<0.001	<0.001	<0.001	–
Anxiety	No^b^	2,571	–	9.9 (8.7–11.0)	–	–	38.2 (36.4–40.1)
	Yes	521	–	31.3 (27.3–35.3)	–	–	70.8 (66.9–74.7)
	*P*		–	<0.001	–	–	<0.001
Sleep problems	No^c^	2,675	13.4 (12.1–14.7)	–	–	–	39.9 (38.1–41.8)
	Yes	417	39.1 (34.4–43.8)	–	–	–	68.1 (63.6–72.6)
	*P*		<0.001	–	–	–	<0.001

† *95% CI, 95% confidence interval*.

a *Higher stress, defined as a total score of Perceived Stress Scale-10 ≥ 14*.

b *Not having anxiety symptoms, defined as a total score of Generalized Anxiety Disorder 7-Item (GAD-7) Scale <5*.

c *Having sleep problems, defined as a total score of Sleep-Rating Scale of Sleep <23*.

The prevalence of sleep problems was significantly higher in college students with the following characteristics: postgraduates (20.0 vs. 12.6%) who read the news with higher frequency daily [None (21.1%); 1–3 times (11.7%); 4–7 times (14.9%); more than 7 times (20.7%)]. Sleep problems were increased among those who had a higher frequency of going out per week [None (12.3%); 1–3 times (14.9%); who went out more than 3 times (20.0%)], who went out wearing a mask [not wearing a mask (18.5%); wearing a disposable medical mask (11.7%); wearing an N95 mask or medical protective mask (15.4%); or who went out wearing other common masks (14.6%)]. Of these, a number had anxiety symptoms (31.3 vs. 9.9%) and higher perceived stress (21.0 vs. 7.6%).

The prevalence of any of the two psychological problems was significantly higher in college students with the following characteristics: postgraduates (33.9 vs. 23.8%) and those who read the news with higher frequency daily [None (25.6%); 1–3 times (22.8%); 4–7 times (27.2%); more than 7 times (37.8%)]. Either of these psychological problems was experienced by those who had a higher frequency of going out per week [None (23.3%); 1–3 times (27.1%); more than 3 times (33.8%)], and those who went out not wearing a mask [not wearing a mask (30.4%); wearing a disposable medical mask (23.4%); N95 mask or medical protective mask (24.9%); or other common masks (27.1%)], and there was a higher perceived level of stress (38.0 vs. 15.0%).

The prevalence of both of the two psychological problems was significantly higher in college students with the following characteristics: postgraduates (8.5 vs. 4.8%) and those who read the news with higher frequency daily [None (5.3%); 1–3 times (4.0%); 4–7 times (6.7%); more than 7 times (12.5%)]. Both problems were experienced by those who went out wearing a mask [not wearing a mask (10.1%); disposable medical mask (3.8%); N95 mask or medical protective mask (5.5%); other common masks (6.7%)], with few remaining masks [0 (9.8%); 1–10 (5.0%); 10–20 (4.1%), more than 20 masks left (4.1%) and those who did not know the number of remaining masks (3.4%)], and there was higher perceived stress (10.3 vs. 1.4%).

The prevalence of higher perceived stress was significantly greater in college students with the following characteristics: those who read the news with higher frequency daily [None (53.4%); 1–3 times (42.7%); 4–7 times (41.9%); more than 7 times (49.7%)], those who went out not wearing a mask [not wearing a mask (54.0%); disposable medical mask (41.4%); N95 mask or medical protective mask (42.5%); other common masks (45.6%)], and those who had few masks [0 (53.5%); 1–10 (41.3%); 10–20 (46.6%); more than 20 (37.7%); those who did not know how many remaining masks they had (52.0%)], those having anxiety symptoms (70.8 vs. 38.2%), and those having sleep problems (68.1 vs. 39.9%).

### Factors Associated With Anxiety Symptoms, Sleep Problems, and Perceived Stress Among College Students

By establishing a logistic regression model, we found that there was a higher frequency of reading the news daily [1–3 times (OR, 2.3, 95%CI: 1.3–4.3); 4–7 times (OR, 3.2, 95%CI: 1.6–6.1); more than 7 times (OR, 5.1, 95%CI: 2.6–9.9)], higher perceived stress (OR, 3.4, 95%CI: 2.8–4.2), and sleep problems (OR, 3.1, 95%CI: 2.4–4.0) were significantly associated with a higher risk of the symptoms of anxiety. Protection factors associated with anxiety symptoms included that they were careless with the number of remaining masks (OR, 0.4, 95%CI: 0.2–0.7) (Table 2).

**Table 2:** Factors associated with anxiety symptoms, sleep problems, and perceived stress among college students^†^.

**Characteristic**	**Group**	**Anxiety symptoms OR (95%CI)**	**Sleep problems OR (95%CI)**	**Both of the two OR (95%CI)**	**Perceived stress OR (95%CI)**
Gender	Male	1	1	1	1
	Female	1.2 (1.0–1.5)	1.0 (0.8–1.3)	1.3 (0.9–1.9)	0.9 (0.8–1.1)
Education	Bachelor	1	1	1	1
	Postgraduate	1.3 (1.0–1.7)	1.6 (1.2–2.1)^**^	1.6 (1.1–2.5)^*^	0.9 (0.7–1.2)
Frequency of daily news reading	None	1	1	1	1
	1–3 times	2.3 (1.3–4.3)^**^	0.5 (0.3–0.8)^**^	1.0 (0.5–2.4)	0.7 (0.5–1.0)
	4–7 times	3.2 (1.6–6.1)^**^	0.6 (0.4–1.1)	2.0 (0.8–4.9)	0.7 (0.4–0.9)^*^
	More than 7 times	5.1 (2.6–9.9)^***^	0.8 (0.4–1.3)	3.3 (1.4–7.9)^**^	0.7 (0.5–1.1)
Frequency of going out per week	None	1	1	1	1
	1–3 times	1.1 (0.9–1.4)	1.3 (1.1–1.7)^*^	1.3 (0.9–1.8)	1.1 (0.9–1.3)
	More than 3 times	1.3 (0.8–2.1)	1.5 (0.9–2.4)	1.6 (0.8–3.2)	1.3 (0.9–1.9)
Mask type	Not wearing a mask	1	1	1	1
	Disposable medical mask	0.7 (0.5–1.0)	1.2 (0.8–1.7)	0.8 (0.5–1.4)	0.9 (0.7–1.2)
	N95 mask or medical protective mask	0.8 (0.6–1.1)	0.9 (0.6–1.1)	0.6 (0.4–0.9)^*^	0.9 (0.7–1.1)
	Other common masks	1.1 (0.7–1.7)	1.2 (0.8–1.9)	1.3 (0.7–2.3)	1.1 (0.8–1.5)
Remaining mask	0	1	1	1	1
	1–10	1.0 (0.7–1.4)	1.0 (0.7–1.4)	0.8 (0.5–1.3)	0.7 (0.5–0.9)^**^
	10–20	0.9 (0.6–1.4)	0.8 (0.5–1.3)	0.6 (0.3–1.2)	0.9 (0.7–1.2)
	More than 20	0.9 (0.6–1.3)	1.0 (0.6–1.5)	0.7 (0.4–1.3)	0.6 (0.5–0.8)^**^
	Careless the number of remaining masks	0.4 (0.2–0.7)^**^	1.2 (0.7–2.1)	0.4 (0.2–1.1)	1.2 (0.8–1.7)
Network behavior	Online games	1	1	1	1
	Others	0.9 (0.7–1.2)	1.0 (0.8–1.3)	0.8 (0.6–1.3)	0.9 (0.7–1.1)
Stress	Normal	1	1	1	–
	Higher^a^	3.4 (2.8–4.2)^***^	2.4 (1.9–3.1)^***^	7.7 (5.0–12.1)^***^	–
Anxiety	No^b^	–	1	–	1
	Yes	–	3.1 (2.4–3.9)^***^	–	3.4 (2.8–4.2)^***^
Sleep problems	No^c^	1	–	–	1
	Yes	3.1 (2.4–4.0)^***^	–	–	2.5 (2.0–3.1)^***^

† *OR, odds ratio; 95%CI,95%confidence interval*.

a *Higher stress, defined as a total score of Perceived Stress Scale-10 ≥ 14*.

b *Not having anxiety symptoms, defined as a total score of Generalized Anxiety Disorder 7-Item (GAD-7) Scale <5*.

c *Having sleep problems, defined as a total score of Sleep-Rating Scale of Sleep <23*.

* *p < 0.05*,

** *p < 0.01*,

*** *p < 0.001*.

Postgraduates (OR, 1.6, 95%CI: 1.2–2.1), the frequency of going out per week (1–3 times) (OR, 1.3, 95%CI: 1.1–1.7), higher perceived stress (OR, 2.4, 95%CI: 1.9–3.1) and having anxiety symptoms (OR, 3.1, 95%CI: 2.4–3.9) were significantly associated with a higher risk of sleep problems. The protection factors associated with sleep problems included the frequency of daily news reading (1–3 times) (OR, 0.5, 95%CI: 0.3–0.8).

Having anxiety symptoms (OR, 3.4, 95%CI: 2.8–4.2) and having sleep problems (OR, 2.5, 95%CI: 2.0–3.1) were significantly associated with a higher risk of perceived stress. Protection factors associated with higher perceived stress included a higher frequency of daily news reading (4–7 times) (OR, 0.7, 95%CI: 0.4–0.9) and remaining masks [1-10 (OR, 0.7, 95%CI: 0.5–0.9); more than 20 (OR, 0.6, 95%CI: 0.5–0.8)].

## Discussion

In this survey, the prevalence of college students with psychological problems was 25.1%: anxiety symptoms 16.8%, sleep problems 13.5%, both of the two 5.3%. 43.7% of college students had perceived higher stress. In this study, the factors associated with anxiety symptoms included sleep problems, stress levels, the number of remaining masks, and the frequency of daily news reading. Factors associated with sleep problems included stress levels, anxiety symptoms, the frequency of going out per week, and daily news reading. The factors associated with perceived stress included anxiety symptoms, sleep problems, the frequency of daily news reading, and the number of remaining masks. The prevalence of anxiety symptoms among college students was significantly higher than during the non-epidemic period in China (7.5%) (20).

The prevalence of anxiety symptoms in college students during the COVID-19 epidemic was higher than that reported in the general population (21). This might be because of the suddenness and unpredictability of the epidemic, which affected normal academic planning. However, the prevalence of anxiety symptoms was slightly lower than that reported in another study involving college students (22). This might be explained by the fact that the epidemic gradually came under control, with a decreasing number of newly confirmed COVID-19 cases during the survey. Besides, unlike the previous study, the population of subjects was investigated in this study to include not only medical students but also non-medical students. Compared with medical students, non-medical students might be less prone to psychological problems (23).

Reflecting the findings of a previous study, college students with higher perceived stress had a higher risk of symptoms of anxiety symptoms and sleep problems. When exposed to chronic stressors in the long-term, people were more likely to have psychological problems such as depression and anxiety (24).

Compared with college students who did not know the number of remaining masks they had, those without any mask had a higher risk of anxiety symptoms. Studies have shown that wearing masks could effectively reduce the risk of contracting the virus (25, 26). Therefore, those without any masks were more prone to anxiety symptoms.

Those who frequently read news about the epidemic had a higher risk of anxiety symptoms. In an “online” society, news spreads faster than before. The long-term impact of receiving constant news about adverse events can have negative psychological effects. Furthermore, the frequency of mobile phone dependence in college students was higher than before (27). During the early stages of the epidemic, unconfirmed news was reprinted on all kinds of social media. Similar to other findings, college students addicted to online games were more likely to report psychological problems (28). Based on these findings, we suggest that colleges should consider issuing guidance and suggestions to regulate the online behaviors of students through official channels, to reduce the impact of unhealthy internet behaviors on physical and psychological well-being.

Sleep problems among college students was a significant factor that can be used to measure their psychological problems. In this study, the prevalence of sleep problems among participants was slightly higher than before the pandemic (29). This might be because the college students that were surveyed were quarantined at home during the outbreak. This experience was accompanied by uncertainty about the development of the epidemic. Compared with those on bachelor degrees, postgraduate students were more prone to sleep problems. This might be because they faced additional pressure from scientific research. College students with a higher frequency of going out per week had a higher risk of having sleep problems. This could be explained by their fear of being infected or going out frequently. In addition to anxiety and insomnia, college students also faced additional pressure. As discussed above, many colleges and Universities delayed the start of school terms due to the epidemic, which might bring further stress. How to intervene in their psychological conditions during and after the epidemic is also a key issue for colleges and Universities to explore.

## Conclusions

The prevalence of anxiety symptoms and sleep problems were high in the investigated college students. The factors associated with anxiety symptoms and sleep problems varied. Bearing in mind the importance of precision prevention, our findings suggest that targeted psychological intervention for college students should be integrated into the work plan to fight against the COVID-19 epidemic.

## Limitations

There are three limitations to this study. Firstly, this study, which was carried out during the COVID-19 epidemic, adopted a non-probability sampling survey, not a random sample survey. Secondly, the survey tools in this study could only be used to evaluate psychological health states instead of psychological disorders. Finally, there were many factors associated with anxiety, sleep, and perceived stress in addition to the impact of COVID-19, and caution should be taken when extrapolating the results.

## Data Availability Statement

The raw data supporting the conclusions of this article will be made available by the authors, without undue reservation.

## Ethics Statement

The research was approved by the Research Ethics Board at The First Affiliated Hospital of Nanchang University and electronic informed consent was obtained from each investigated subject (approval number: 050).

## Author Contributions

XW and LL: formal analysis, investigation, and writing of the original draft. HC: conceptualization, methodology, investigation, reviewing, and editing the manuscript. YL: investigation and writing of the original draft. NZ: formal analysis and investigation. ZS, QL, and WG: investigation. BH and ML: conceptualization, data curation, methodology, investigation, reviewing, editing the manuscript, and supervision. All authors contributed to the article and approved the submitted version.

## Conflict of Interest

The authors declare that the research was conducted in the absence of any commercial or financial relationships that could be construed as a potential conflict of interest.

## References

[1] Meo SA Alhowikan AM Al-Khlaiwi T Meo IM Halepoto DM Iqbal M Novel coronavirus 2019-nCoV: prevalence, biological and clinical characteristics comparison with SARS-CoV and MERS-CoV. Eur Rev Med Pharmacol Sci. (2020) 24:2012–19. 10.26355/eurrev_202002_20379 32141570

[2] Johns Hopkins University. COVID-19 Dashboard by the Center for Systems Science and Engineering (CSSE) at Johns Hopkins University (JHU). (2020). Retrieved from https://gisanddata.maps.arcgis.com/apps/opsdashboard/index.html#/bda7594740fd40299423467b48e9ecf6/ (accessed May 31, 2020).

[3] Wang CY Pan RY Wan XY Tan YL Xu LK Ho Cyrus S. Immediate psychological responses and associated factors during the initial stage of the 2019 coronavirus disease (COVID-19) epidemic among the general population in China. Int J Environ Res Public Health. (2020) 17:1729. 10.3390/ijerph17051729 32155789 PMC7084952

[4] United Nations Educational Scientific and Cultural Organization (UNESCO) COVID-19 Education Disruption and Response. (2020). Retrieved from https://en.unesco.org/covid19/educationresponse/ (accessed May 31, 2020).

[5] Qiu JY Shen B Zhao M Wang Z Xie B Xu YF. A nationwide survey of psychological distress among Chinese people in the COVID-19 epidemic: implications and policy recommendations. Gen Psychiatry. (2020) 33:e100213. 10.1136/gpsych-2020-100213 32215365 PMC7061893

[6] Golberstein E Wen HF Miller Benjamin F. Coronavirus disease 2019 (COVID-19) and mental health for children and adolescents. JAMA Pediatr. (2020) 174:819–820. 10.1001/jamapediatrics.2020.1456 32286618

[7] Kang LJ Li Y Hu SH Chen M Yang C Yang BX. The mental health of medical workers in Wuhan, China dealing with the 2019 novel coronavirus. Lancet Psychiatry. (2020) 7:e14. 10.1016/S2215-0366(20)30047-X 32035030 PMC7129673

[8] Zhai YS Du X. Mental health care for international Chinese students affected by the COVID-19 outbreak. Lancet Psychiatry. (2020) 7:e22. 10.1016/S2215-0366(20)30089-4 32199511 PMC7103995

[9] Davenport MH Meyer S Meah VL Strynadka MC Khurana R Moms are not ok: COVID-19 and maternal mental health. Front Glob Womens Health. (2020) 1:1 10.3389/fgwh.2020.00001 PMC8593957 34816146

[10] Yang Y Li W Zhang QG Zhang L Teris Cheung Xiang YG. Mental health services for older adults in China during the COVID-19 outbreak. Lancet Psychiatry. (2020) 7:e19. 10.1016/S2215-0366(20)30079-1 32085843 PMC7128970

[11] Zhai YS Du X. Addressing collegiate mental health amid COVID-19 pandemic. Psychiatry Res. (2020) 288:113003. 10.1016/j.psychres.2020.113003 32315885 PMC7162776

[12] Chen HG Zhang KL. Insight into the psychological problems on the epidemic of COVID-19 in China by online searching behaviors. J Affect Disord. (2020) 276:1093–94. 10.1016/j.jad.2020.07.128 32771861 PMC7395660

[13] Spitzer RL Kroenke K Williams Janet BW Löwe Bernd. A brief measure for assessing generalized anxiety disorder: the GAD-7. Arch Intern Med. (2006) 166:1092–97. 10.1001/archinte.166.10.1092 16717171

[14] Richardson T Wrightman M Yeebo M Lisicka A Reliability and score ranges of the PHQ-9 and GAD-7 in a primary and secondary care mental health service. J Psychosoc Rehabil Ment Health. (2017) 4:237–40. 10.1007/s40737-017-0090-0

[15] Cohen S Kamarck T Mermelstein R. A global measure of perceived stress. J Health Soc Behav. (1983) 24:385–96. 10.2307/2136404 6668417

[16] Lu Wei Bian Q Wang WZ Wu XL Wang Z Zhao M. Chinese version of the perceived stress scale-10: a psychometric study in Chinese university students. PLoS ONE. (2017) 12:e0189543. 10.1371/journal.pone.0189543 29252989 PMC5734731

[17] Li JM Yin SF Duan JX Zhang QB Analysis rating of sleep state of 13273 normal persons. Health Psychol J. (2000) 8:351–53.

[18] Li JM Self-rating scale of sleep. Chin J Health Psychol. (2012) 020:1851.

[19] Meng XZ Ding K Liu XM An empirical study of reliability and validity of self-rating scale of sleep in maintenance of stability soldiers. World J of Sleep Med. (2015) 2:241−44.

[20] Kirwan M Pickett SM Jarrett NL Emotion regulation as a moderator between anxiety symptoms and insomnia symptom severity. Psychiatry Res. (2017) 254:40–47. 10.1016/j.psychres.2017.04.028 28448803

[21] Ren YL Zhou YJ Qian W Li ZZ Liu ZK Wang RX. Letter to the editor “A longitudinal study on the mental health of general population during the COVID-19 epidemic in China.” *Brain Behav Immun*. (2020) 87:132–33. 10.1016/j.bbi.2020.05.004 32387510 PMC7201232

[22] Chang JH Yuan YX Wang D. Mental health status and its influencing factors among college students during the epidemic of COVID-19. Nan Fang Yi Ke Da Xue Xue Bao. (2020) 40:171–76. 10.12122/j.issn.1673-4254.2020.02.06 32376528 PMC7086131

[23] Li YC Wang Y Jiang JW Valdimarsdottir UA Fall K Fang F. Psychological distress among health professional students during the COVID-19 outbreak. Psychol Med. (2020) 11:1–3. 10.1017/S0033291720001555 32389148 PMC7225209

[24] Schneiderman N Ironson G Siegel SD. Stress and health: psychological, behavioral and biological determinants. Annu Rev Clin Psychol. (2005) 1:607–28. 10.1146/annurev.clinpsy.1.102803.144141 17716101 PMC2568977

[25] Ma QX Shan H Zhang HL Li GM Yang RM Chen JM. Potential utilities of mask-wearing and instant hand hygiene for fighting SARS-CoV-2. J Med Virol. (2020) 92:1567–71. 10.1002/jmv.25805 32232986 PMC7228401

[26] Eikenberry SE Mancuso M Iboi E Phan T Eikenberry K Kuang Y To mask or not to mask: modeling the potential for face mask use by the general public to curtail the COVID-19 pandemic. Infect Dis Model. (2020) 5:293–308. 10.1016/j.idm.2020.04.001 32355904 PMC7186508

[27] Mei SL Chai JX Wang SB Ng CH Ungvari GS Xiang YT. Mobile phone dependence, social support and impulsivity in chinese University students. Int J Environ Res Public Health. (2018) 15:504. 10.3390/ijerph15030504 29533986 PMC5877049

[28] Mohammadi B Szycik GR Te WB Heldmann M Samii A Munte TF. Structural brain changes in young males addicted to video-gaming. Brain Cogn. (2020) 139:105518. 10.1016/j.bandc.2020.105518 31954233

[29] Ye Y Du GH Fan F Li LY Guo QG Chen SJ A comparison of sleep quality and influencing factors between young soldiers and college students. Chin J Clin Psychol. (2013) 2:306.

